# Bacterial Community and Genomic Analysis of Carbapenem-Resistant *Acinetobacter baumannii* Isolates from the Environment of a Health Care Facility in the Western Region of Saudi Arabia

**DOI:** 10.3390/ph15050611

**Published:** 2022-05-16

**Authors:** Muhammad Yasir, Abdullah Mohammad Subahi, Hani A. Shukri, Fehmida Bibi, Sayed Sartaj Sohrab, Maha Alawi, Anees A. Sindi, Asif A. Jiman-Fatani, Esam I. Azhar

**Affiliations:** 1 Special Infectious Agents Unit, King Fahd Medical Research Center, King Abdulaziz University, Jeddah 21589, Saudi Arabia; a-subahi@hotmail.com (A.M.S.); fehmeedaimran@yahoo.com (F.B.); sayedsartaj@gmail.com (S.S.S.); eazhar@kau.edu.sa (E.I.A.); 2 Department of Medical Laboratory Sciences, Faculty of Applied Medical Sciences, King Abdulaziz University, Jeddah 21589, Saudi Arabia; 3 Clinical and Molecular Microbiology Laboratories, King Abdulaziz University Hospital, Jeddah 21589, Saudi Arabia; hanishukri@yahoo.com (H.A.S.); afatani@kau.edu.sa (A.A.J.-F.); 4 Department of Medical Microbiology and Parasitology, Faculty of Medicine, King Abdulaziz University, Jeddah 21589, Saudi Arabia; dr_mma1@yahoo.com; 5 Infection Control & Environmental Health Unit, King Abdulaziz University Hospital, King Abdulaziz University, Jeddah 21589, Saudi Arabia; 6 Department of Anesthesia and Critical Care, Faculty of Medicine, King Abdulaziz University, Jeddah 21589, Saudi Arabia; asindi2@kau.edu.sa

**Keywords:** *Acinetobacter baumannii*, hospital environment, genome, bacteria, antimicrobial-resistance, Saudi Arabia

## Abstract

The escalating transmission of hospital-acquired infections, especially those due to antimicrobial-resistant bacteria, is a major health challenge worldwide. In this study, a culturomic analysis of bacterial community in a tertiary care hospital in the western region of Saudi Arabia is performed using environmental samples. The genome sequencing of four *Acinetobacter baumannii* was performed on isolates recovered from an intensive care unit (ICU) environment and clinical samples. A total of 361 bacterial isolates from surface and air samples were identified by MALDI-TOF technique or 16S rRNA gene sequencing. The isolates were classified into 70 distinct species, including ESKAPE pathogens. Resistance in Gram-positive isolates was mainly found to be against benzylpenicillin, azithromycin, ampicillin, and trimethoprim/sulfamethoxazole. Carbapenem- and multidrug-resistant isolates of *A. baumannii* and *Klebsiella pneumonia* were found on the ICU surfaces. Genome sequencing revealed that the carbapenem-resistant *A. baumannii* isolate from ICU environment was linked with those of clinical origin. The isolate Ab133-HEnv was classified as a novel sequence type (ST2528) based on a new allele of Oxf_gdhB-286. Three beta-lactam-antibiotic-resistance genes, *bla*_ADC-25_, *bla*_OXA-23_, and *bla*_OXA-66_, were found in most of the analyzed genomes. Collectively, the results of this study highlight the spread of antimicrobial-resistant nosocomial pathogens in a health care facility in Saudi Arabia.

## 1. Introduction

Hospital-acquired infections (HAIs) due to multidrug-resistant (MDR) bacteria are a serious safety concern for patients and health care providers [1,2,3]. Based on a survey of more than 50 hospitals in Southeast Asia, Europe, Western Pacific, and Eastern Mediterranean countries, the World Health Organization (WHO) estimated an average 8% prevalence of HAIs [4]. The incidence of HAIs in the United States and in European countries ranges between 13.0 and 20.3 episodes per 1000 patient days [5]. A literature review revealed a fragmented picture of the endemic burden of HAIs in low- and middle-income countries [5], with an overall prevalence of 5.7% to 19.1% and a pooled prevalence of 10.1% [5]. The risk of HAIs is markedly high in intensive care units (ICUs), with approximately 30% of patients experiencing an episode of HAI, resulting in significant morbidity and mortality [6]. The annual financial loss from HAIs has been estimated to be EUR 7 billion in Europe and USD 6.5 billion in the United States [5].

Numerous factors contribute to the acquisition of HAIs, including contaminated instruments, close contact with infected patients or health care providers, and environmental contamination within the hospital [1]. Invasive medical devices, such as urinary catheters, intravascular cannulas, and ventilator tubing, are the main sources of nosocomial pathogens [1,7]. Based on the screening of a hospital environment, Lemmen et al. confirmed the microbial contamination of equipment, patient bed spaces, clinical areas, and other general sites [8]. *Acinetobacter baumannii*, *Staphylococcus aureus*, *Clostridium difficile*, *Pseudomonas aeruginosa*, and *Enterococcus* species are among the microorganisms that can survive in hospital environments for long periods of time [7,8,9]. The most common HAIs are pneumonia, urinary tract infections, bloodstream infections, and surgical site infections [1]. Cleaning all areas around the patient is vital for the prevention of HAIs.

*Acinetobacter baumannii* is certainly one of the most successful pathogens responsible for HAI in the modern healthcare system and with increasing reports of community-acquired infections [3,10,11]. The mortality rates from this pathogen range from 8% to 35%, depending on the type of infection and the duration of the hospital stay [12]. Most healthcare-associated infections caused by *A. baumannii* involve ventilator-associated pneumonia and bloodstream infections. The mortality rates of these infections range from 5% in hospital wards to 54% in ICUs [13]. *Acinetobacter baumannii* carries multiple survival mechanisms under a wide range of environments and the ability to develop multidrug resistance [14]. This bacterium has a remarkable capacity to survive on abiotic surfaces, from non-medical objects, such as door knobs, sinks, bed rails, and pillows, to medical equipment, such as ventilator circuits and catheters [3,15,16]. As a consequence, *A. baumannii* is transmitted through contact with inanimate objects, making it a constant threat to old, critically ill, and immunosuppressed patients [12,13].

The growing evidence of extensively drug-resistant and pandrug-resistant isolates of *A. baumannii* is also accumulating in the different geographical regions of the world [3,9,15]. Approximately 70% of *A. baumannii* isolates in the Middle East are considered to have multidrug resistance [15,17]. In particular, carbapenem resistance is increasing among *A. baumannii* strains and is considered to be a main cause of HAIs, leading to high mortality, especially in ICUs [10]. About 1 million cases of *A. baumannii* infections occur globally per year, and half of them are resistant to multiple antibiotics, including carbapenems [18]. Surveillance reports show that more than 50% of *A. baumannii* from the US and European ICUs are carbapenem-resistant [19]. WHO has classified carbapenem-resistant *A. baumannii* as a critical priority pathogen posing a serious threat to human health and urgently needed to make new antibiotics available [20]. Additionally, HAIs surveillance is essential for detecting *A. baumannii* outbreaks and containing them [1,12]. Although the standard methods used for typing during outbreak surveillance are helpful in revealing relationships between isolates, they are ineffective for distinguishing between closely related strains. The whole-genome sequencing method has proven to be more accurate for separating closely related strains, and it can also provide a comprehensive picture of antimicrobial resistance genes (ARGs) and virulence factors [21,22].

The available literature highlights that HAIs represent a critical issue in the health care facilities of Saudi Arabia [9,23,24]. A study from Abha General Hospital showed that 14% of the patients admitted to neonatal intensive care units acquired a nosocomial infection [25]. Zowawi et al. reported a high prevalence of extended-spectrum β-lactamase and carbapenemase-producing Gram-negative bacteria commonly linked with HAIs in the Arabian Peninsula [26]. Overall, surveillance studies for nosocomial pathogens and antimicrobial resistance (AMR) are necessary to detect newly emerging resistance and provide appropriate management support for infection control in health care facilities. In the current study, a hospital microbiome screening program is initiated at a major tertiary care hospital in the western region of Saudi Arabia to monitor microbial community and AMR trends in the hospital environment and to compare them with isolates that cause HAI. Further, *A. baumannii* isolates recovered from hospital environments, and clinical isolates are characterized by genome sequencing.

## 2. Results

### 2.1. Bacterial Community Analysis

**Figure 1:**
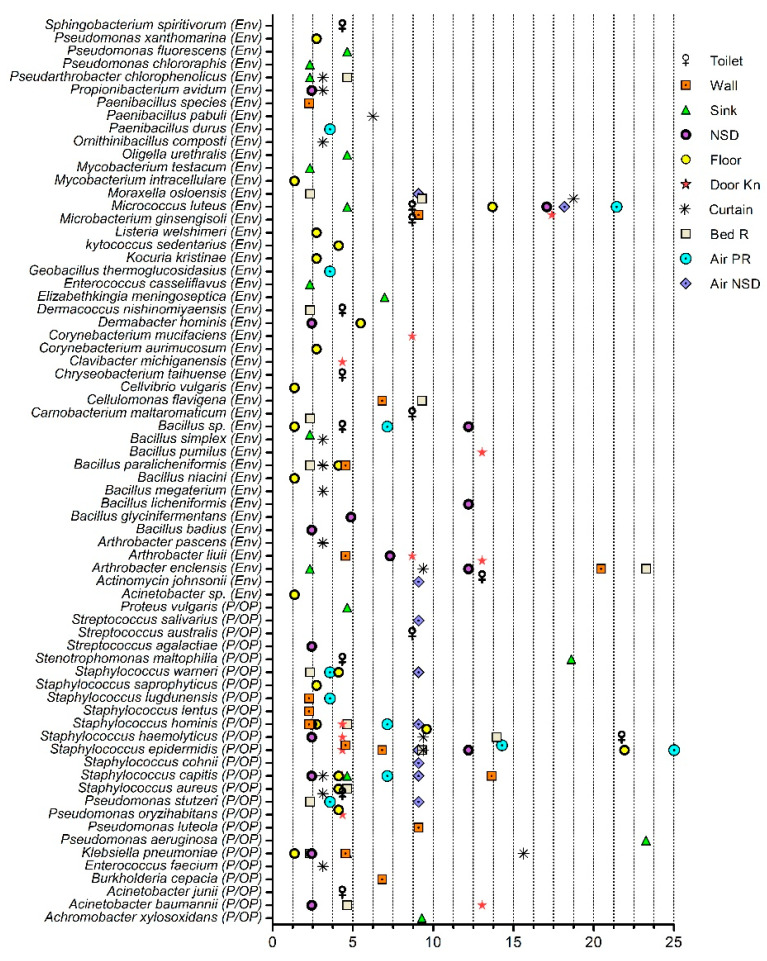
Distribution of environmental, pathogenic, and opportunistic pathogenic bacteria in the surface and air samples of the hospital units and nurses’ station desks. The *x*-axis represents percentage abundance. Env, environmental; P/OP, pathogenic and opportunistic pathogen; NSD, nurses station desk; Door Kn, doorknob; Bed R, bed rail; Air PR, air patient room; Air NSD, air nurses station desk.

From the hospital environment, 361 isolates were identified and predominantly comprised of Firmicutes followed by Actinobacteria, Proteobacteria, and Bacteroidetes. The isolates were classified into 70 distinct species from 36 genera. By Gram staining, 276 isolates from 47 distinct species were Gram-positive, and 85 isolates from 23 distinct species were Gram-negative. An average of 40.2 ± 15.9 isolates per sample were identified from the surface sites and 19.5 ± 12.0 isolates per air sample. A total of 22 species were identified from 73 isolates of floor samples, followed by 16 species identified from each surface types of sink (43 isolates), curtain (32 isolates), and bed rail (43 isolates), respectively. Isolates were classified into environmental and pathogenic/opportunistic pathogenic bacteria based on the species identification. The majority of the 45 distinct species from 183 isolates were of environmental origin (Figure 1). *Micrococcus luteus* and *Arthrobacter enclensis* were commonly found in the surface samples from the ICUs and the oncology ward of the hospital (Table S1). *Micrococcus luteus* isolates were found at a relatively higher percentage abundance in the floor samples, while *A. enclensis* isolates were found at a relatively high abundances in the bed rail and wall samples (Figure 1). Half of the environmental species were not commonly found in the hospital environment and were found on specific types of surfaces (Figure 1).

#### Pathogenic and Opportunistic Pathogenic Bacteria

Notably, 49.3% (*n* = 178) isolates from the hospital environment were classified into 25 pathogenic/opportunistic pathogenic (P/OP) bacterial species (Figure 1). Among them, 118 isolates from 14 species were Gram-positive, and 60 isolates from 11 species were Gram-negative. On average, 19.1 ± 10.9 P/OP isolates were found on the surface sites, and 12.5 ± 7.8 P/OP isolates were grown from the air samples. Among the ESKAPE pathogens, *Klebsiella pneumoniae* isolates were recovered from six surface sites, with a relative abundance from 2.3% to 15.6% (wall, sink, nurse station desk, floor, curtain, and bed rail; Figure 1). *Staphylococcus aureus* isolates were recovered from the floor and bed rail samples at a <5.0% relative abundance. *Pseudomonas aeruginosa* isolates were recovered from the sink samples (23.5% relative abundance), and *A*. *baumannii* was found on the doorknobs (13.0%), bed rails (4.7%), and nursing station desk (2.4%). *Enterococcus faecium* isolates were found in curtain samples (15.6% relative abundance; Figure 1). Among the other opportunistic bacteria, *Staphylococcus haemolyticus* was found at a relatively high abundance (14.0–21.7%) on toilet seats, bed rails, and air samples from the patients’ rooms. *Staphylococcus epidermidis* was found at relatively higher abundances in the air samples from the patient rooms, floor, and nurse station desk samples (12.2–25%; Figure 1). 

### 2.2. Phenotypic Antimicrobial Resistance

**Figure 2:**
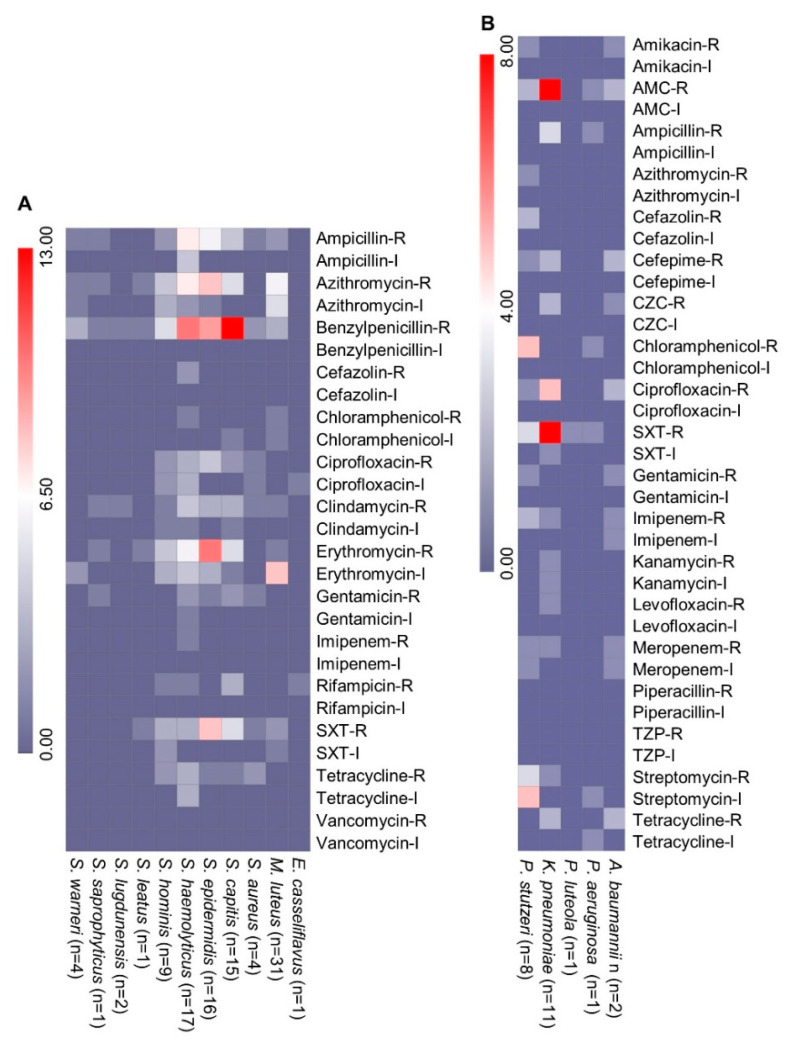
Antimicrobial susceptibility of the isolates from the hospital environment. (**A**) Antimicrobial susceptibility of Gram-positive bacteria and (**B**) Gram-negative bacteria. The scale is based on the number of isolates. R, resistant; I, intermediately resistant; SXT, trimethoprim/sulfamethoxazole; AMC, amoxicillin/clavulanic acid; TZP, piperacillin/tazobactam; CZC, ceftazidime/clavulanic acid; n, number of isolates.

A total of 124 isolates were tested for antimicrobial susceptibility, and 101 Gram-positive isolates from 11 species were screened against 14 clinically important antibiotics. A maximum of 49 isolates were resistant or intermediately resistant to erythromycin, followed by benzylpenicillin (48), azithromycin (45), ampicillin (28), and trimethoprim/sulfamethoxazole (26). None of the tested isolates was resistant to vancomycin (Figure 2A). The lowest resistance was found in <10 isolates against the antibiotics imipenem and cefazolin, followed by chloramphenicol, rifampicin, and gentamycin. From the HAI-associated pathogens, two *S. aureus* isolates were MDR and had resistance to benzylpenicillin, tetracycline, gentamicin, ciprofloxacin, and trimethoprim/sulfamethoxazole. The coagulase-negative *S*. *haemolyticus* isolates had variable resistance to the tested antibiotics, and five of the isolates were MDR (Figure 2A). The isolates were mainly resistant to benzylpenicillin, ampicillin, azithromycin, erythromycin, and clindamycin. Half of the coagulase-negative *S. epidermidis* (8/16) isolates were MDR and were mainly resistant to benzylpenicillin, erythromycin, trimethoprim/sulfamethoxazole, azithromycin, and ciprofloxacin. Among the 15 isolates of *Staphylococcus capitis*, 5 isolates were MDR and mainly resistant to benzylpenicillin, erythromycin, trimethoprim/sulfamethoxazole, and azithromycin. Among other Gram-positive bacteria, four MDR isolates of *Staphylococcus hominis* were found on the different surface sites (Figure 2A).

From Gram-negative bacteria, 23 isolates from five P/OP species were tested against 19 antibiotics (Figure 2B). Among the 11 *K. pneumonia* isolates, 6 were MDR and were mainly resistant to trimethoprim/sulfamethoxazole, amoxicillin/clavulanic acid, and ciprofloxacin (Figure 2B). The MDR isolate Kp4-HEnv isolated from the medical intensive care unit (MICU) curtain sample was resistant to carbapenems. Among other Gram-negative bacteria, two MDR isolates of *A. baumannii* and one isolate of *Pseudomonas stutzeri* were recovered. The *P. stutzeri* isolate Ps92-HEnv was isolated from MICU and was resistant to 11 tested antibiotics. The MDR *A. baumannii* Ab27-HEnv was isolated from a patient bed railing in MICU, and Ab133-HEnv was isolated from the nurses’ station desk. The isolate *A. baumannii* Ab27-HEnv was resistant to carbapenems. 

WHO has classified carbapenem-resistant *A. baumannii* amongst the global priority list of antibiotic-resistant bacteria posing a serious threat to human health and the main cause of HAIs [20]. The further genomic analysis of the AMR isolates of *A. baumannii* was performed recovered from the hospital environment and clinical specimens of the patients of the same health care facility.

### 2.3. Genomic Analysis of A. baumannii Isolates

Next, *A. baumannii* isolates from the clinical samples of patients and the hospital environment were analyzed (Table S2). Two of the isolates, including Ab43 recovered from a bronchial wash and Ab91 recovered from a wound sample, had almost the same phenotype pattern of AMR as Ab127-HEnv performed on VITEK-2 with a specific antibiotic susceptibility card for Gram-negative bacteria (Table 1). Three of the isolates were resistant to carbapenem, ampicillin, third- and fourth-generation cephalosporin (ceftazidime and cefepime), piperacillin/tazobactam, ciprofloxacin, gentamycin, and tobramycin (Table 1). In contrast, Ab133-HEnv was sensitive to all the tested antibiotics except cefepime and ciprofloxacin (Table 1). 

The genome characterization of the isolates from the hospital environment and clinical isolates from the relevant ICU patients is summarized in Table 1. The genome analysis revealed that Ab27-HEnv, Ab43, and Ab91 belong to ST218 based on the Oxford MLST scheme and to ST2 based on the Pasteur MLST scheme. A new allele type of Oxf_gdhB-286 was found in Ab133-HEnv and was classified as a novel ST2528 following the Oxford MLST scheme. Based on the Pasteur MLST scheme, the new allele was assigned as fusA-405 and a novel ST2089. Annotation data obtained from the PATRIC annotation server revealed that the draft genomes contained 3904, 3439, 3822, and 3783 coding sequences in Ab27-HEnv, Ab133-HEnv, Ab43, and Ab91, respectively (Table 1). A synteny map of the whole-genome alignment with the reference genome *A. baumannii* ATCC 19606 showed a lesser ratio of genome gaps in the Ab133-HEnv strain as compared with the genomes of the other three isolates. The map shows the size of all genomes with the greatest size being the Ab43 genome and a larger number of gaps in the Ab27-HEnv genome (Figure S1). The clinical isolates Ab43 and Ab91 were closely linked and made a clade with Ab27-HEnv, whereas Ab133-HEnv made a clade with the reference isolate genome ATCC 19606 in a dendrogram created based on the synteny map (Figure S1).

#### Antimicrobial Resistance Genes and Mobile Genetic Elements Analysis

**Figure 3:**
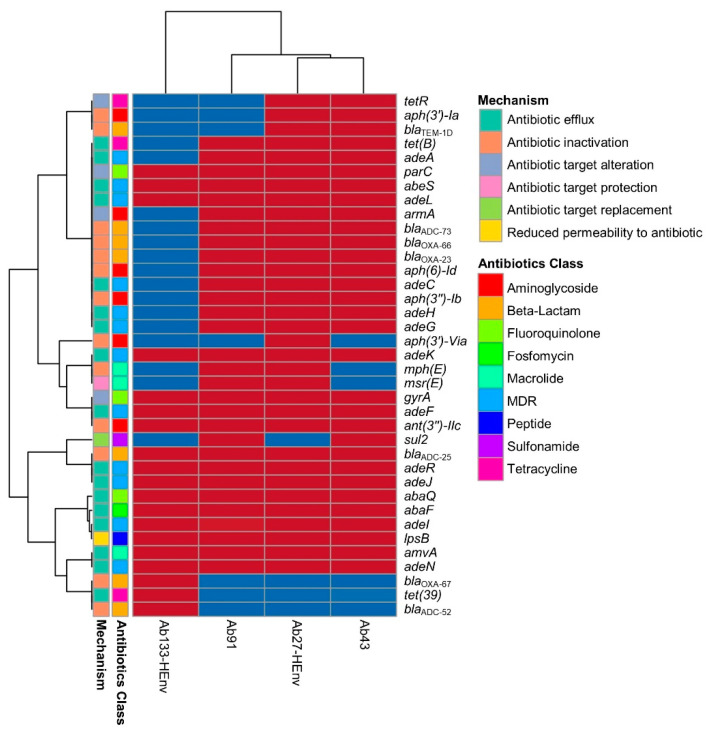
Acquired and intrinsic resistance genes retrieved from the *Acinetobacter baumannii* genomes of the isolates recovered from the hospital environment and clinical samples. MDR, multidrug resistance. The blue box indicates the respective gene was not detected.

The genomic analysis identified 19 to 33 ARGs in the four sequenced isolates, producing resistance against mainly eight classes of antibiotics and the genes associated with multidrug resistance (Figure 3). Among the non-acquired 17 antibiotic resistance-associated efflux genes, 12 were common among the genomes of the four *A. baumannii* isolates. In the Ab27-HEnv isolate, 12 acquired resistance genes were found, and 7 of them were found in the clinical isolates (Ab43 and Ab91), including the resistance genes for carbapenem (*bla*_OXA-23_), other beta-lactam antibiotics (*bla*_ADC-25_, *bla*_OXA-66_), aminoglycoside (*aph(3″)-Ib, aph(6)-Id*, and *armA*), and tetracycline (*tetB*). In addition, the Ab27-HEnv isolate was carrying *mphE*, *bla*_TEM-1D_, and *msrE* genes, which produce resistance against macrolide and beta-lactam and multidrug resistance, respectively (Figure 3). In the second isolate from the hospital environment, Ab133-HEnv, a minimum number of acquired resistance genes was detected, producing resistance against beta-lactam antibiotics (*bla*_ADC-25_ and *bla*_OXA-67_) and tetracycline (*tet39*). The *bla*_OXA23_ gene was detected on the separate contig in the three genomes Ab27-HEnv, Ab43, and Ab91 and could be of plasmid origin, which was confirmed by the mlplasmids v2.1.0 tool.

Seven mobile genetic elements (MGEs) were found in the Ab27-HEnv isolate, including five insertion sequences and two composite transposons. Three of the MGEs carrying contigs had resistance genes for aminoglycoside (*aph(6)-Id, aph(3″)-Ib*, *aph(3′)-Via*, and *armA*) and macrolide (*mph*E) antibiotics. Moreover, the insertion sequences IS*26*, IS*Vsa3*, IS*Aba24*, and IS*Aba26* were also found in the clinical isolates. All four isolates were identified as human pathogens with a probability of >85% based on carrying pathogenic family member genes.

### 2.4. Comparative Analysis of A. baumannii Genomes from Genbank 

#### 2.4.1. Acquired AMR Genes Analysis

**Figure 4:**
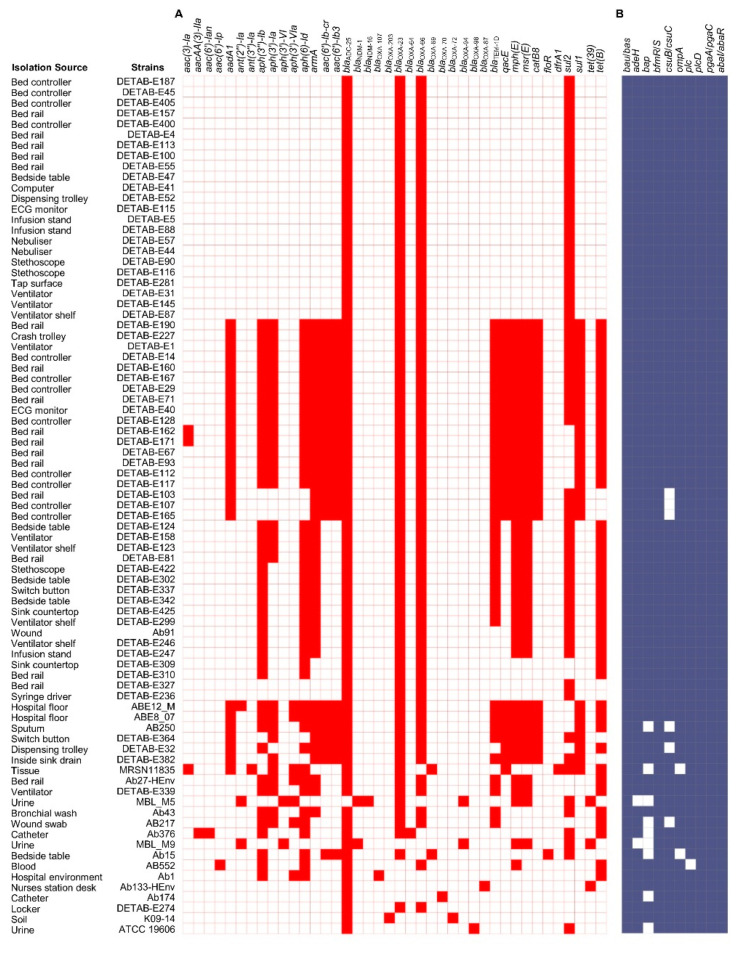
Genomic analysis of antimicrobial resistance genes (ARGs) and virulence factors. (**A**) The pattern of acquired resistance genes and (**B**) virulence factors associated genes in the *A. baumannii* genomes. The white box indicates the respective gene was not detected.

To understand the AMR pattern in the *A. baumannii* isolates recovered from the hospital environment, the acquired resistance genes in 81 *A. baumannii* genomes were analyzed. These genomes represented 72 isolates from hospital environments, 7 from clinical samples, and 2 reference isolates. An average of 10.0 ± 5.7 acquired ARGs were found in the 72 *A. baumannii* genomes recovered from the hospital environment. In the clinical isolates, an average of 10.6 ± 3.8 ARGs were found, whereas the reference strain ATCC 19606 initially isolated in 1948 carried 3 resistance genes, *bla*_ADC-25_, *bla*_OXA-98_, and *sul*2 (Figure 4A). Three of the beta-lactam antibiotics resistance genes, *bla*_ADC-25_, *bla*_OXA-23_, and *bla*_OXA-66_, were found in ≥87% of the analyzed genomes. The seven ARGs for aminoglycoside (*aph(6)-Id, aph(3″)-Ib*, and *armA*), sulfonamide (*sul2*), macrolide (*mphE*), tetracycline (*tetB*), and the MDR gene (*msrE*) were commonly found in ≥50 genomes (Figure 4A). The insertion sequence regions mainly of IS*Aba1* were located in the proximity of the *bla*_OXA-23_ and *bla*_ADC-25_ genes in most of the genomes carrying these resistance genes contigs (Table S3). The main resistance pattern based on four ARGs (*bla*_ADC-25_, *bla*_OXA-23_, *bla*_OXA-66_, and *sul*2) was detected in 23 isolates of the hospital environment (Figure 4A; Table S2). The second key pattern of 18 ARGs was observed in 10 isolates recovered from the hospital environment (Figure 4A). The ARG pattern was unique in the genome sequences from this study as well as other *A. baumannii* genomes from Saudi Arabia retrieved from GenBank, with the exception of Ab91, which carried 10 ARGs that were previously found in two genomes of ICU isolates recovered from a ventilator shelf, and an infusion stand in China (Figure 4A).

#### 2.4.2. Virulence Factor Genes and Phylogenetic Analysis

**Figure 5:**
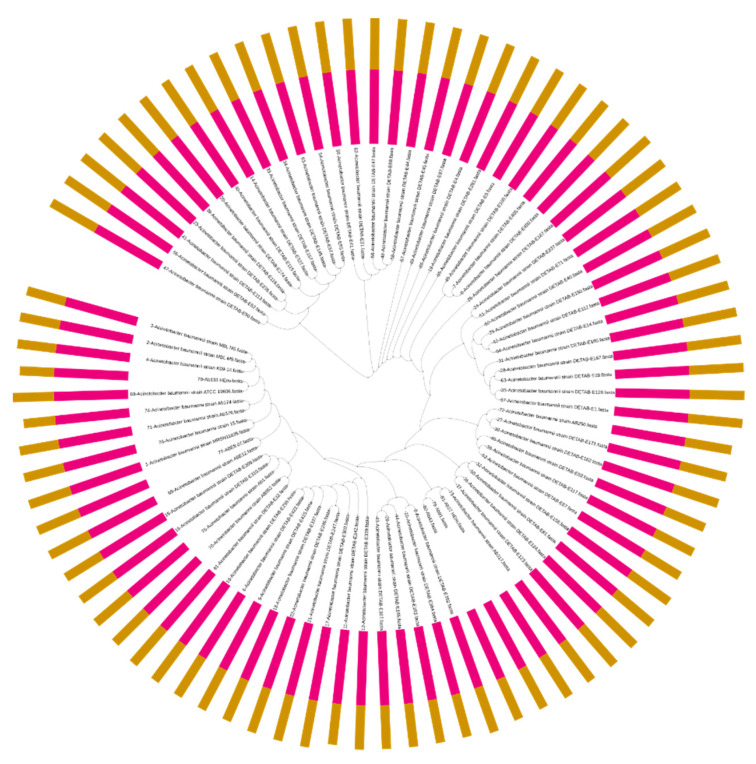
Multilocus-typing- and pangenome-based phylogenetic analysis. The pink bars around the tree represent the fixed number of core genes, and the yellow type bars show the variating number of accessory genes.

No substantial variation was found in the genes associated with virulence factors in the *A. baumannii* genomes (Figure 4B). The genes associated with the factors acinetobactin (*bau*/*bas*), two-component system (*bfmR*/S), phospholipase D (*plcD*), serum resistance (*pbpG*), and quorom sensing (*abaI*/*abaR*) were commonly found in all 81 genomes, and other genes, including those encoding the AdeFGH efflux pump (*adeG*), biofilm-associated protein (*bap*), pili (*csuB*/*csuC*), outer membrane protein gene (*ompA*), and phospholipase C (*plc*), were found in >80% of the genomes (Figure 4B). The phylogenetic analysis based on an MLST and pan-genome estimation analysis predicted the sequences of 2177 core proteins shared by all 81 genomes. These 81 genomes contained a total of 113,090 accessory genes, which have evolved at a faster rate than the core genes. These genes, along with homologs in sister clades, also showed an accelerated rate of evolution among the isolates compared with the core genome. Moreover, 23 genomes have a sum of 1450 unique genes present in their pan-genomes, showing the specificity to those strains. The phylogenetic tree revealed the existence of nine well-supported monophyletic groups belonging to related clonal complexes (Figure 5).

## 3. Discussion

Bacterial species in the hospital environment make patients susceptible to HAIs, with immunocompromised patients, postsurgical patients, patients with invasive medical devices, and patients undergoing chemotherapy being especially at risk [1,27]. It is important to regularly screen hospital environments to document the hospital microbiome and to identify potential sources of HAIs. The rate of HAIs changes according to the studied patient population, surveillance techniques, and hospital settings (e.g., hospital wards), and HAIs are relatively common in ICUs [1,28]. In this study, we screened three ICUs and an oncology ward in a tertiary care hospital in the western region of Saudi Arabia, using a culture-based technique to identify nosocomial infection-associated P/OP bacteria. A total of 70 species from 36 genera were identified from the surface and air samples of the hospital sites, and they mainly comprised Gram-positive isolates belonging to *Staphylococcus* and *Micrococcus* genera. Gram-negative isolates were found at relatively lower abundances in the hospital environment and were mainly from the genera *Pseudomonas* and *Klebsiella*. The HAI-associated pathogens, such as coagulase-negative staphylococci (CoNS), *K. pneumoniae*, *P. aeruginosa*, *A. baumannii*, *Stenotrophomonas maltophilia*, *S. aureus*, and *E. faecium*, were found on different hospital surfaces in this study. CoNS are considered normal inhabitants of the human skin and mucous membranes [29]. CoNS are also recognized as prevalent Gram-positive nosocomial bacteria and a common cause of bloodstream infection, followed by *S. aureus* at a surgical site [29]. Consistent with our findings, Johani et al. predominantly found *Staphylococcus* followed by *Propionibacterium*, *Bacillus*, *Enterococcus*, and *Streptococcus* as common genera among Gram-positive bacteria in adults and children ICUs at a tertiary care hospital in Jeddah, Saudi Arabia [30]. 

Previously, a relatively high abundance of HAIs from Gram-negative bacteria, such as *E. coli*, *Klebsiella* species, and *P. aeruginosa*, was reported in the multi-hospital health care system in Saudi Arabia [23]. These nosocomial pathogens have the ability to colonize medical devices and hospital environment, and they can cause high morbidity and mortality in surgery, oncology, hematology, burn, and ICU patients [1,27,31]. Infections caused by these virulent bacteria are difficult to control and treat because of their increasing resistance to many antimicrobial agents [23]. In this study, a relatively low percentage of *P. aeruginosa* isolates were identified. However, several *K. pneumoniae* isolates, including MDR isolates, were identified from the hospital surface samples. *Klebsiella pneumoniae* is responsible for many types of HAIs, including skin and soft tissue infections, septicemia, urinary tract infections, and pneumonia. It is associated with hospital-acquired outbreaks due to its ability to spread rapidly in hospital environments [32,33]. *A. baumannii* is a ubiquitous pathogen that can cause hospital-acquired and community-acquired infections [16]. Because of its ability to accumulate mechanisms of drug resistance that lead to pan-AMR and cause large hospital-acquired outbreaks that often involve multiple facilities [3,16]. *Acinetobacter baumannii* mainly causes bloodstream, urinary tract, pulmonary, and surgical wound infections [16]. In this study, we recovered two *A. baumannii* isolates from MICU and nursing station desk surface samples. *Stenotrophomonas maltophilia* is another Gram-negative organism that causes HAIs, and it has also been linked with hospital-acquired outbreaks [34]. It can infect patients who have underlying medical conditions, including low immune status, as well as intubated patients, and it causes skin and soft tissue infection, bacteremia, urinary tract infection, and endocarditis [34]. Similar to other Gram-negative bacteria, *S. maltophilia* was detected at a lower percentage in this study. We found high proportions of some bacteria, such as *Micrococcus* species and *Bacillus* species, but their chance of causing infection is minimal. Similarly, a study from Brazil found *Streptococcus*, *Propionibacterium*, *Micrococcus*, and *Staphylococcus* to be common genera in the hospital environment [35]. Pereira et al. observed a lower abundance of Gram-negative bacteria and mainly isolated *Salmonella enterica* and *K. pneumoniae* from frequently touched surfaces in a teaching hospital in São Paulo [35].

Many factors can interfere with the control of nosocomial infections, notably the specific types of bacteria and their resistance to antimicrobial agents. Physicians face difficulty finding effective treatments to treat infections from MDR bacteria in chronic care facilities [7,23,36]. Important antibiotics such as penicillin, semi-synthetic penicillin, and first- and second-generation cephalosporin are losing efficacy [7,37]. It is crucial to not only check the hospital microbiome, but also the antibiotic susceptibility of the bacteria isolated. In the current study, the disk diffusion method using Mueller–Hinton medium was used for assessing antimicrobial susceptibility. Only clinically relevant isolates were processed for antimicrobial susceptibility and the ones for which a breakpoint was available from CLSI guidelines and compatible with growing on Mueller–Hinton-medium plates. The Gram-negative bacteria isolated in this study were most susceptible to the carbapenem antibiotics imipenem and meropenem. Moreover, the Gram-positive isolates in this study were also susceptible to the tested carbapenem antibiotics. Consistent with this study, other reports from Saudi Arabia showed that high numbers of ESBL isolates were susceptible to carbapenem antibiotics [38,39]. In addition, a retrospective study of the data collected from the National Nosocomial Infections Surveillance System between 1986 and 2003 showed a significantly increased resistance to carbapenem and aminoglycoside with time [2]. Still, carbapenem antibiotics are considered the ultimate choice for treating infections by ESBL-positive pathogens [2,40]. 

In this study, most of the tested isolates recovered from the hospital environment were susceptible to aminoglycoside. Gentamicin has been used to treat serious Gram-negative bacterial infections since the early 1960s, and it remains a first-line treatment [41]. Based on our study, carbapenem and aminoglycoside showed good inhibitory activity against the isolates from the hospital environment. In addition, we observed low resistance against tetracycline in both Gram-positive and Gram-negative isolates. However, other studies have reported increased resistance to tetracycline, especially through the mechanism of acquiring resistance genes [42]. Around 70% of isolates from this study were susceptible to ciprofloxacin from the quinolone group. Low resistance was observed against cefazolin from the cephalosporin group. Cephalosporin antibiotics have a wide range of antibacterial activity against Gram-positive and Gram-negative bacteria, but they show considerable diversity in their antibacterial properties [39,43,44]. A study from the Netherlands showed a shift of antibiotic usage to cephalosporin in combination with metronidazole and gentamicin due to better results as a prophylactic among ICU patients [43]. However, another study showed an increased incidence of cefepime resistance in Gram-negative pathogenic bacteria [24,39]. Comparatively higher resistance to sulfonamide was observed in the Gram-negative isolates in this study than in the Gram-positive isolates. Co-trimoxazole is used clinically for treating infections of the respiratory tract and urinary tract and for treating other infections caused by Gram-negative and Gram-positive bacteria. Moreover, a recent study showed that the association of *sul1* and *sul2* resistance genes with MGEs probably contributes to the increasing resistance against sulfonamide [45]. In the Gram-positive isolates, high resistance was observed against benzylpenicillin, consistent with the trend observed globally [46]. Consistent with previous studies, the most effective antibiotic against Gram-positive isolates in this study was vancomycin [47].

Over the past two decades, *A. baumannii* has emerged as one of the most successful nosocomial pathogens because of its ability to survive in hospital environments for prolonged periods and its rapid acquisition of resistance to an extensive range of antimicrobial agents [3,14]. Previous studies have shown that *A. baumannii* could be isolated from medical apparatuses, bed rails, water systems, and sinks in the hospital, notably from ICUs and surgical wards [3,14,16]. In this study, *A. baumannii* isolates were recovered from a bed railing and the nursing station desk in the MICU. Patients and health care workers frequently touch these surfaces. Based on MLST analysis, the isolate Ab27-HEnv was linked with two clinical isolates, Ab43 and Ab91, recovered from clinical samples of isolation ward and MICU patients, respectively. The cross-contamination of pathogenic bacteria among ICU surfaces, health care workers, and patients has been reported in several studies [22,48]. The high occupancy rate and heavy workload in ICUs increase transmission risk. 

HAIs caused by carbapenem-resistant *A. baumannii* result in substantial morbidity and mortality, a serious health threat, and an economic burden worldwide [3,16]. *Acinetobacter baumannii* resistant to carbapenems, with or without the *armA* gene, has previously been reported from hospitals in Saudi Arabia and the Gulf region [9,24,49,50]. In this study, the majority of the analyzed genomes harbored the *bla_OXA-23_* and *bla_OXA66_* genes, which are responsible for carbapenem resistance. The *bla_OXA-23_* gene was detected in the *A. baumannii* isolates of this study that were phenotypically resistant to meropenem and imipenem [51]. The results are consistent with previous studies showing that *bla*_OXA-23_ is the most widely reported gene, while *bla*_OXA66_ is the most common OXA variant in *A. baumanni*i [9,24,50]. In *A. baumannii*, the augmented antibiotic resistance is largely attributed to the actions of MGEs and the activation of intrinsic resistance mechanisms, such as chromosomal β-lactamases, *bla*_ADC_, and efflux pump activity [3,14,52]. In addition, diverse MGEs were found in the Ab27-HEnv isolate. It is noteworthy that insertion sequences, such as IS*26*, IS*Vsa3*, IS*Aba24*, and IS*Aba26*, and a composite transposon were retrieved in the *A. baumannii* genome. The AdeFGH RND efflux pumps were previously identified in isolates with carbapenem resistance [52]. According to other reports, these genetic structures could facilitate the spread of antibiotic resistance determinants among pathogens, potentially compromising the effectiveness of many antibiotic treatments, including those prescribed to patients [3,52]. Furthermore, the carbapenem-resistant isolates were also phenotypically resistant to several antibiotics often used in clinical practice.

Virulence factors, in conjunction with drug resistance, are widely believed to be responsible for the ability of *A. baumannii* to grow in unfavorable conditions, particularly in the hospital environment [52,53]. Interestingly, the genes associated with virulence factors, such as biofilm formation, immune evasion, iron uptake, phospholipases, and adherence, were conserved in most of the genomes analyzed in this study. The presence of the virulence genes *csu*ABCD, *bfmR*/S, *entE*, *ompA*, and *plcD* suggests the infectious nature of these strains [52,53]. Csu pili (*csuA*/BABCDE, which are regulated by the *bfmR*/*S* two-component system), biofilm-associated proteins (BAP), and poly-N-acetylglucosamine production (*pga*ABCD) are all involved in biofilm formation, maintenance, and maturation [11]. Studies have found that biofilm formation promotes the survival of *A. baumannii* strains on surface sites in hospital environments [11]. The presence of the most significant virulence genes in the hospital environment isolates aligns with their clinical importance and strengthens their association with HAIs [11,16].

## 4. Materials and Methods

### 4.1. Sample Collection

**Figure 6:**
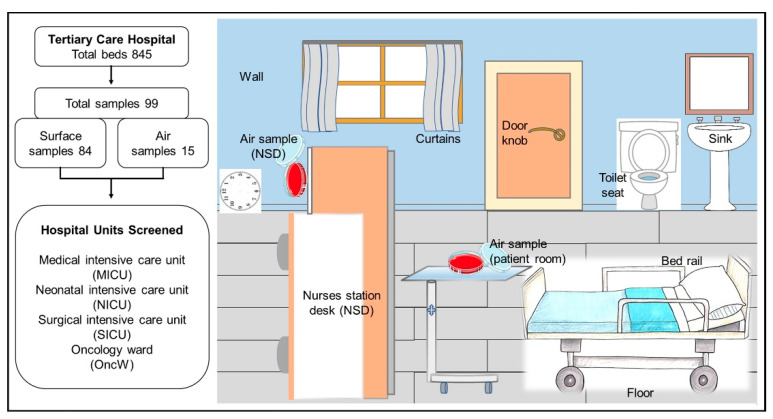
Overview of the sampling sites. The diagram shows the various surface sites from the hospital units that were sampled.

This study was approved by the ethics committee of the Faculty of Medicine at King Abdulaziz University (reference no. 235-15). The environmental samples were collected from a large 845-bed tertiary care hospital in the western region of Saudi Arabia. A total of 84 swab samples and 15 air samples were collected from seven rooms in the medical intensive care unit (MICU), two rooms in the neonatal intensive care unit (NICU), four rooms in the surgical intensive care unit (SICU), and three rooms in the oncology ward (OncW). With the exception of one SICU room, the rooms were occupied by patients at the sampling time. Sampling sites included frequently touched surfaces, patients’ surroundings, common areas, and nurse desk stations (Figure 6). These sites were selected based on previous studies suggesting that such sites can pose a high risk of nosocomial pathogen transmission [15,22,48]. Sterile swabs moistened with sterile normal saline (0.85%) were streaked across 25 cm^2^ of large surfaces and surfaces smaller than 25 cm^3^ were swabbed in their entirety. The portion of the swab in contact with the collector’s hand was removed, and the remaining portion was placed in a sterile tube containing 1 mL of sterile normal saline. Air samples were collected using sheep blood agar plates that were left open on a table inside each room or at the nurse stations for 1 h (Figure 6) [54]. During sampling, collectors wore personal protective equipment, such as gloves, a facemask, and a disposable gown.

### 4.2. Identification and Antimicrobial Susceptibility Screening

Swab samples were shaken in 10 mL of sterile normal saline at 200 RPM for 30 min. Samples were serially diluted, and 100 µL from each dilution was spread on three types of media (sheep blood agar, Colombia agar, and MacConkey agar). Plates were incubated at 37 °C for 48 h, and colonies were purified by subculturing. Isolates were identified using the high-throughput MALDI-TOF-based VITEK-MS (BioMérieux, Marcy-l’Étoile, France) system described previously [55]. The calibration was performed using the reference *Escherichia coli* ATCC 25922 strain to validate the run. A percentage probability of 99.9% in comparison with database spectra was used for correct identification. Unidentified isolates from MALDI-TOF were processed for identification using 16S rRNA gene sequence analysis. The 16S rRNA gene was amplified by PCR from extracted DNA of each isolate using universal primers set 27f and 1492r, as described previously [56]. Purified PCR products were sequenced with an ABI Prism Sequencer 3730 (Applied Biosystems, Waltham, MA, USA) according to the manufacturer’s instructions. Sequences were BLAST searched in the EzTaxon database for the identification of the bacteria (https://www.ezbiocloud.net/ accessed on 10 March 2022).

Antimicrobial susceptibility was determined using the standard disk diffusion assay. In brief, isolates were cultured overnight on plates with their respective medium. Suspensions from fresh cultures were prepared by emulsifying the isolates in 0.45% saline to the equivalent of a 0.5 McFarland turbidity standard by measuring with a DensiCheck instrument (BioMérieux, Marcy-l’Étoile, France). Each isolate suspension was spread on Mueller–Hinton medium agar, and antibiotic disks were placed on the agar. After 20 h of aerobic incubation at 37 °C, inhibition zones were measured. Zone sizes were interpreted according to the guidelines of the Clinical and Laboratory Standards Institute [57]. Selective antibiotics were used against Gram-positive and Gram-negative bacteria (Table S4).

### 4.3. Genome Sequencing and Data Analysis

Genome sequencing was performed for the four *A. baumannii* isolates, including two isolates recovered from the MICU and nursing station desk, respectively, and two recovered from clinical samples from patients admitted in the same healthcare facility as previously reported [24]. Briefly, the clinical isolate Ab43 was recovered from the bronchial wash of a 52-year-old patient diagnosed with acquired immunodeficiency syndrome admitted to the isolation ward. The isolate Ab91 was recovered from a wound specimen of 73-year-old patient that was admitted to the MICU. Antimicrobial activity and minimum inhibitory concentration of the isolates against a panel of 13 antibiotics were assessed using VITEK 2 (BioMérieux, France) with a specific antimicrobial susceptibility card against Gram-negative bacteria (AST-N291). DNA was extracted from the four isolates using a bacterial DNA extraction kit (Norgen Biotek Corp., Thorold, ON, Canada), and genome sequencing was performed as described previously [44]. A Nextera XT DNA library preparation kit (Illumina, Inc., San Diego, CA, USA) was used to prepare libraries, and sequencing was performed with a V3 kit, using 2 × 300 bp chemistry on the MiSeq platform (Illumina, Inc., USA). The quality assessment of the raw sequence reads was performed using FastQC2, and the sequences were trimmed using the Trimmomatic v0.32 tool. The contig assemblies were prepared with the SPAdes 3.15.3 program. 

In addition, we retrieved 77 genomes of *A. baumannii* from the GenBank database, mainly recovered from the hospital environment and reference genomes of *A. baumannii* ATCC 19606 and *A. baumannii* K09-14 (Table S2). Genome annotation was performed using PATRIC 3.6.12. Multilocus sequence typing (MLST) analysis was performed using PubMLST (https://pubmlst.org/ accessed on 12 March 2022). Pan-genome analysis was performed using the pipeline of BPGA v1.3 [58]. USEARCH was used in the BPGA v1.3 workflow to find orthologous protein clusters using a 0.5 cutoff. Paralogs were excluded from the genomes, and protein sequences were extracted from orthologous gene clusters. A phylogenetic tree was constructed to understand the genetic diversity of the sequenced isolates based on MLST and pan-genome estimation analysis that predicted the sequences of 2177 core proteins shared by all 81 genomes. ARGs were identified using ResFinder3.1 and CARD (Comprehensive Antibiotic Resistance Database, Hamilton, ON, Canada) [59,60]. The MobileElementFinder v1.0.3, NCBI nucleotide BLAST, and ISfinder tools were used to determine the mobile genetic elements (MGEs). The blast criteria of bit scores above 50 and E. value of lower than 1^−10^ was used to consider the presence of insertion elements in the BLAST search. The genomes assemblies were searched for plasmid contigs using the mlplasmids v2.1.0 tool [61]. Virulence-associated genes were retrieved and annotated using Virulence Factor Database (http://www.mgc.ac.cn/VFs/ accessed on 24 March 2022). Genome sequences were deposited into NCBI GenBank under the accession numbers JAKJQQ000000000–JAKJQT000000000.

## 5. Conclusions

Overall, the cultured bacterial community of the tertiary care hospital in the western region of Saudi Arabia comprised diverse bacterial species, mainly of environmental bacteria. P/OP bacteria associated with HAIs were also found in surface and air samples from ICUs, oncology wards, and nursing stations. We identified carbapenem-resistant and virulent strains of *A. baumannii* from the MICU environment that exhibited genotypes matching those of clinical isolates from patients treated at the same site, highlighting the importance of environmental hygiene. Genomic analysis confirmed the distribution of carbapenem resistance-associated genes in *A. baumannii* isolates recovered from the environment in other health care facilities. Frequent surveillance for pathogens and AMR in the hospital environment is recommended for data-driven infection control measures to effectively combat HAIs.

## Figures and Tables

**Table 1:** Antimicrobial susceptibility and genomic annotation of the *Acinetobacter baumannii* isolates.

Features	Ab27-HEnv	Ab133-HEnv	Ab43	Ab91
Phenotypic Resistance				
Ciprofloxacin	R	R	R	R
Gentamicin	R	S	R	R
Tobramycin	R	S	R	R
Ampicillin	R	S	R	R
Cefepime	R	R	R	R
Ceftazidime	R	S	R	R
Ceftriaxone	S	S	S	I
Imipenem	R	S	R	R
Meropenem	R	S	R	R
TZP	R	S	R	R
Tigecycline	S	S	I	I
SXT	S	S	R	R
Minocycline	R	S	R	I
Genomic features				
MLST (Oxford)	ST218	ST2528	ST218	ST218
MLST (Pasture)	ST2	ST2089	ST2	ST2
Contigs	75	21	150	65
GC Content	38.87	39.03	39.01	38.92
Contig L50	9	2	22	9
Contig N50	131,103	838,947	64,059	165,021
CDS	3904	3439	3822	3783
tRNA	64	63	59	57
rRNA	4	4	5	3
Hypothetical proteins	1007	671	938	930
Proteins with functional assignments	2897	2768	2884	2853
Proteins with EC number assignments	938	931	939	937
Proteins with GO assignments	806	799	808	806
Proteins with pathway assignments	726	728	728	728
Proteins with PATRIC genus-specific family (PLfam) assignments	3720	3352	3651	3646
Proteins with PATRIC cross-genus family (PGfam) assignments	3841	3385	3765	3730

R, resistant; I, intermediately resistant; S, susceptible; TZP, piperacillin/tazobactam; SXT, trimethoprim/sulfamethoxazole; MLST, multilocus sequence typing; CDS, coding sequences.

## Data Availability

Genome sequences were deposited into NCBI GenBank under the accession numbers JAKJQQ000000000–JAKJQT000000000.

## Supplementary Materials

The following supporting information can be downloaded at: https://www.mdpi.com/article/10.3390/ph15050611/s1, Figure S1: The synteny map is showing whole genome alignment of the *Acinetobacter baumannii* from this study with a reference isolate genome of *A. baumannii* ATCC 19606; Tables S1: Percentage distribution of bacterial community in the different units of the hospital; Table S2: Description of the *Acinetobacter baumannii* genomes analyzed in this study; Table S3: List of insertion sequences on the specific beta-lactamase resistance genes carrying contigs in the *Acinetobacter baumannii* genomes assemblies; Table S4: List of antibiotics disk used against Gram-negative and Gram-positive isolates for antibiotic susceptibility screening.
