# Supplementary material for: Bacterial Community and Genomic Analysis of Carbapenem-Resistant Acinetobacter baumannii Isolates from the Environment of a Health Care Facility in the Western Region of Saudi Arabia
Source: Pharmaceuticals (Basel). 2022 May 16;15(5):611. doi: 10.3390/ph15050611 (PMC9145440; doi:10.3390/ph15050611)
Supplement: Supplementary file 1 [file pharmaceuticals-15-00611-s001.zip › pharmaceuticals-1695063-supplementary.pdf]

Supplementary Data

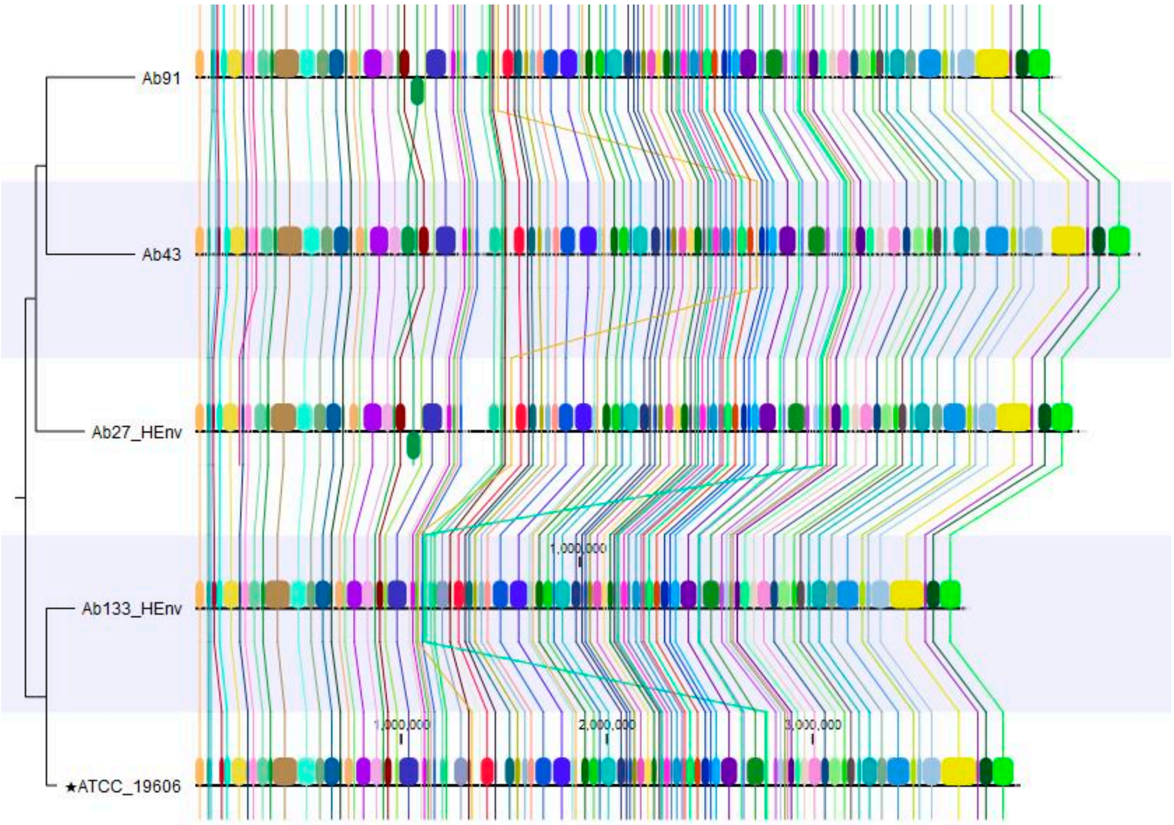

Figure S1. The synteny map is showing whole genome alignment of the *Acinetobacter baumannii* from this study with a reference isolate genome of *A. baumannii* ATCC 19606.

Table S1. Percentage distribution of bacterial community in the different units of the hospital.

Species	MICU	NICU	SICU	OncW
Achromobacter xylosoxidans	2.39521			
Acinetobacter baumannii	1.197605	9.756098		
Acinetobacter junii				1.234568
Acinetobacter sp.				1.234568
Actinomyces johnsonii				1.234568
Arthrobacter enclensis		21.95122	19.44444	13.58025
Arthrobacter liuii			9.722222	
Arthrobacter pascens		2.439024		
Bacillus badius	0.598802			
Bacillus glycinifermentans				2.469136
Bacillus licheniformis	2.994012			
Bacillus megaterium	0.598802			
Bacillus niacini		2.439024		
Bacillus paralicheniformis			5.555556	3.703704
Bacillus pumilus	1.796407			
Bacillus simplex	0.598802			
Bacillus sp.	3.592814	7.317073	2.777778	
Burkholderia cepacia		7.317073		
Carnobacterium maltaromaticum				2.469136
Cellulomonas flavigena			5.555556	3.703704
Cellvibrio vulgaris				1.234568
Chryseobacterium taihuense				1.234568
Clavibacter michiganensis				1.234568
Corynebacterium aurimucosum	1.197605			
Corynebacterium mucifaciens			2.777778	
Dermabacter hominis	2.39521		1.388889	
Dermacoccus nishinomiyaensis	0.598802			1.234568
Elizabethkingia meningoseptica	1.197605		1.388889	
Enterococcus casseliflavus		2.439024		
Enterococcus faecium			1.388889	
Geobacillus thermoglucosidasius	0.598802			
Klebsiella pneumoniae	7.185629			
Kytococcus sedentarius	1.796407			
Listeria welshimeri			2.777778	
Microbacterium ginsengisoli				2.469136
Microbacterium testaceum				1.234568
Micrococcus luteus	13.17365	17.07317	11.11111	12.34568
Moraxella osloensis		2.439024	1.388889	
Mycobacterium avium				1.234568
Oligella urethralis	1.197605			

Ornithinibacillus composti				1.234568
Paenibacillus durus			1.388889	
Paenibacillus pabuli	1.197605			
Paenibacillus species	0.598802			
Propionibacterium avidum			1.388889	1.234568
Proteus vulgaris	1.197605			
Pseudarthrobacter chlorophenolicus		2.439024	1.388889	2.469136
Pseudomonas aeruginosa	3.592814		5.555556	
Pseudomonas chlororaphis		2.439024		
Pseudomonas fluorescens		4.878049		
Pseudomonas luteola	2.39521			
Pseudomonas oryzihabitans		2.439024		
Pseudomonas stutzeri	3.592814	2.439024		1.234568
Pseudomonas xanthomarina				2.469136
Rothia kristinae	1.197605			
Sphingobacterium spiritivorum				1.234568
Staphylococcus aureus	2.994012			
Staphylococcus capitis	7.185629	2.439024	1.388889	3.703704
Staphylococcus cohnii				1.234568
Staphylococcus epidermidis	17.36527		5.555556	8.641975
Staphylococcus haemolyticus	9.580838		2.777778	13.58025
Staphylococcus hominis	2.994012	7.317073	2.777778	
Staphylococcus lentus	0.598802			
Staphylococcus lugdunensis				2.469136
Staphylococcus saprophyticus				2.469136
Staphylococcus warneri	0.598802	2.439024	2.777778	2.469136
Stenotrophomonas maltophilia	1.796407		6.944444	1.234568
Streptococcus agalactiae			1.388889	
Streptococcus australis				2.469136
Streptococcus salivarius			1.388889	

MICU, medical intensive care unit; NICU, neonatal intensive care unit; SICU, surgical intensive care unit; OncW, oncology ward.

Table S2. Description of the *Acinetobacter baumannii* genomes analyzed in this study.

Accession No.	Strains	Isolation Source	Geographic Location	Genome Source
GCA_021725575.1	Ab27-HEnv	Bed rail	Saudi Arabia	This study
GCA_021726495.1	Ab133-HEnv	Nurses station desk	This study	This study
GCA_021726525.1	Ab43	Bronchial wash	This study	This study
GCA_021725595.1	Ab91	Wound	This study	This study
GCA_002245665.1	Ab15	Bedside table	Brazil	GenBank
GCA_002760765.1	MBL_M5	Urine	Tunisia	GenBank
GCA_002760845.1	MBL_M9	Urine	Tunisia	GenBank
GCA_012524275.1	Ab376	Catheter	Argentina	GenBank
GCA_020101315.1	DETAB-E405	Bed controller	China	GenBank
GCA_020101595.1	DETAB-E422	Stethoscope	China	GenBank
GCA_020101775.1	DETAB-E44	Nebuliser	China	GenBank
GCA_020101795.1	DETAB-E52	Dispensing trolley	China	GenBank
GCA_020101815.1	DETAB-E47	Bedside table	China	GenBank
GCA_020101835.1	DETAB-E45	Bed controller	China	GenBank
GCA_020101875.1	DETAB-E55	Bed rail	China	GenBank
GCA_020101935.1	DETAB-E57	Nebuliser	China	GenBank
GCA_020102055.1	DETAB-E67	Bed rail	China	GenBank
GCA_020102315.1	DETAB-E88	Infusion stand	China	GenBank
GCA_020102355.1	DETAB-E93	Bed rail	China	GenBank
GCA_020102395.1	DETAB-E90	Stethoscope	China	GenBank
GCA_020102415.1	DETAB-E87	Ventilator shelf	China	GenBank
GCA_020102555	DETAB-E1	Ventilator	China	GenBank
GCA_020102615.1	DETAB-E100	Bed rail	China	GenBank
GCA_020102635.1	DETAB-E103	Bed rail	China	GenBank
GCA_020102715.1	DETAB-E107	Bed controller	China	GenBank
GCA_020102755.1	DETAB-E113	Bed rail	China	GenBank
GCA_020102935.1	DETAB-E124	Bedside table	China	GenBank
GCA_020102995.1	DETAB-E115	ECG monitor	China	GenBank
GCA_020103075.1	DETAB-E116	Stethoscope	China	GenBank
GCA_020103215.1	DETAB-E14	Bed controller	China	GenBank
GCA_020103455.1	DETAB-E145	Ventilator	China	GenBank
GCA_020103655.1	DETAB-E158	Ventilator	China	GenBank
GCA_020103835.1	DETAB-E162	Bed rail	China	GenBank
GCA_020103895.1	DETAB-E160	Bed rail	China	GenBank
GCA_020103915.1	DETAB-E167	Bed controller	China	GenBank
GCA_020103935.1	DETAB-E165	Bed controller	China	GenBank
GCA_020104255.1	DETAB-E190	Bed rail	China	GenBank
GCA_020104975.1	DETAB-E227	Crash trolley	China	GenBank
GCA_020105275.1	DETAB-E246	Ventilator shelf	China	GenBank
GCA_020105435.1	DETAB-E247	Infusion stand	China	GenBank
GCA_020105835.1	DETAB-E274	Locker	China	GenBank
GCA_020105975.1	DETAB-E29	Bed controller	China	GenBank

GCA_020106075.1	DETAB-E281	Tap surface	China	GenBank
GCA_020106275.1	DETAB-E31	Ventilator	China	GenBank
GCA_020106295.1	DETAB-E302	Bedside table	China	GenBank
GCA_020106415.1	DETAB-E4	Bed rail	China	GenBank
GCA_020106515.1	DETAB-E309	Sink countertop	China	GenBank
GCA_020106535.1	DETAB-E310	Bed rail	China	GenBank
GCA_020106845.1	DETAB-E337	Switch button	China	GenBank
GCA_020107095.1	DETAB-E342	Bedside table	China	GenBank
GCA_020107135.1	DETAB-E339	Ventilator	China	GenBank
GCA_020107555.1	DETAB-E364	Switch button	China	GenBank
GCA_020108075.1	DETAB-E5	Infusion stand	China	GenBank
GCA_020108135.1	DETAB-E400	Bed controller	China	GenBank
GCA_020108155.1	DETAB-E171	Bed rail	China	GenBank
GCA_020108555.1	DETAB-E71	Bed rail	China	GenBank
GCA_020109495.1	DETAB-E425	Sink countertop	China	GenBank
GCA_020109615.1	DETAB-E157	Bed rail	China	GenBank
GCA_020109735.1	DETAB-E187	Bed controller	China	GenBank
GCA_020109835.1	DETAB-E32	Dispensing trolley	China	GenBank
GCA_020110475.1	DETAB-E123	Ventilator shelf	China	GenBank
GCA_020110515.1	DETAB-E128	Bed controller	China	GenBank
GCA_020110955.1	DETAB-E40	ECG monitor	China	GenBank
GCA_020110975.1	DETAB-E41	Computer	China	GenBank
GCA_020111875.1	DETAB-E299	Ventilator shelf	China	GenBank
GCA_020111935.1	DETAB-E382	Inside sink drain	China	GenBank
GCA_020112215.1	DETAB-E81	Bed rail	China	GenBank
GCA_020112895.1	DETAB-E112	Bed controller	China	GenBank
GCA_020113755.1	DETAB-E117	Bed controller	China	GenBank
GCA_020113775.1	DETAB-E327	Bed rail	China	GenBank
GCA_020114155.1	DETAB-E236	Syringe driver	China	GenBank
GCA_900117405.1	ABE12_M	Hospital floor	Morocco	GenBank
GCA_900119395.1	ABE8_07	Hospital floor	Morocco	GenBank
GCA_016538365.2	MRSN11835	Tissue	USA	GenBank
GCF_001640065.1	AB217	Wound swab	Saudi Arabia	GenBank
GCF_001640075.1	AB250	Sputum	Saudi Arabia	GenBank
GCF_002212105.1	AB552	Blood	Saudi Arabia	GenBank
SAMEA5593850	Ab174	Catheter	Saudi Arabia	GenBank
GCA_009035845.1	ATCC-19606	Urine	USA	GenBank
GCF_008632635	K09-14	Soil	Malaysia	GenBank
GCA_000981405.1	Ab1	Hospital environment	Not Applicable	GenBank

Table S3. List of insertion sequences on the specific beta-lactamase resistance genes carrying contigs in the *Acinetobacter baumannii* genomes assemblies.

Genomes accession No.	Strains	OXA-23 contig		OXA-66 contig		ADC-25 contig	
		bla_{OXA-23} gene	IS	bla_{OXA-66} gene	IS	bla_{ADC-25} gene	IS
GCA_021725575.1	Ab27-HEnv	+	IS*Aba1*	+	-	+	IS*Aba1*
GCA_021726495.1	Ab133-HEnv	-	-	-	-	+	-
GCA_021726525.1	Ab43	+	IS*Aba1*	+	-	+	IS*Aba1*
GCA_021725595.1	Ab91	+	IS*Aba1*	+	-	+	IS*Aba26*
GCA_002245665.1	Ab15	+	IS*Aba1*	-	-	+	IS*Aba1*
GCA_002760765.1	MBL_M5	+	-	-	-	-	-
GCA_002760845.1	MBL_M9	-	-	-	-	+	-
GCA_012524275.1	Ab376	+	IS*Aba1*	-	-	+	IS*Aba1*
GCA_020101315.1	DETAB-E405	+	IS*Aba1*	+	IS*Aba1*	+	IS*Aba1*
GCA_020101595.1	DETAB-E422	+	IS*Aba1*	+	-	+	IS*Aba1*
GCA_020101775.1	DETAB-E44	+	IS*Aba1*	+	-	+	IS*Aba1*
GCA_020101795.1	DETAB-E52	+	IS*Aba1*	+	-	+	IS*Aba1*
GCA_020101815.1	DETAB-E47	+	IS*Aba1*	+	-	+	IS*Aba1*
GCA_020101835.1	DETAB-E45	+	IS*Aba1*	+	-	+	IS*Aba1*
GCA_020101875.1	DETAB-E55	+	IS*Aba1*	+	-	+	IS*Aba1*
GCA_020101935.1	DETAB-E57	+	IS*Aba1*	+	-	+	IS*Aba1*
GCA_020102055.1	DETAB-E67	+	IS*Aba1*	+	-	+	IS*Aba1*
GCA_020102315.1	DETAB-E88	+	IS*Aba1*	+	-	+	IS*Aba1*
GCA_020102355.1	DETAB-E93	+	IS*Aba1*	+	-	+	IS*Aba1*
GCA_020102395.1	DETAB-E90	+	IS*Aba1*	+	-	+	IS*Aba1*
GCA_020102415.1	DETAB-E87	+	IS*Aba1*	+	-	+	IS*Aba1*
GCA_020102555	DETAB-E1	+	IS*Aba1*	+	-	+	IS*Aba1*
GCA_020102615.1	DETAB-E100	+	IS*Aba1*	+	-	+	IS*Aba1*
GCA_020102635.1	DETAB-E103	+	IS*Aba1*	+	-	+	IS*Aba1*
GCA_020102715.1	DETAB-E107	+	IS*Aba1*	+	-	+	IS*Aba1*
GCA_020102755.1	DETAB-E113	+	IS*Aba1*	+	-	+	IS*Aba1*
GCA_020102935.1	DETAB-E124	+	IS*Aba1*	+	-	+	IS*Aba1*
GCA_020102995.1	DETAB-E115	+	IS*Aba1*	+	-	+	IS*Aba1*
GCA_020103075.1	DETAB-E116	+	IS*Aba1*	+	-	+	IS*Aba1*
GCA_020103215.1	DETAB-E14	+	IS*Aba1*	+	-	+	IS*Aba1*
GCA_020103455.1	DETAB-E145	+	IS*Aba1*	+	-	+	IS*Aba1*
GCA_020103655.1	DETAB-E158	+	IS*Aba1*	+	-	+	IS*Aba1*
GCA_020103835.1	DETAB-E162	+	IS*Aba1*	+	-	+	IS*Aba1*
GCA_020103895.1	DETAB-E160	+	IS*Aba1*	+	-	+	IS*Aba1*
GCA_020103915.1	DETAB-E167	+	IS*Aba1*	+	-	+	IS*Aba1*
GCA_020103935.1	DETAB-E165	+	IS*Aba1*	+	-	+	IS*Aba1*

GCA_020104255.1	DETAB-E190	+	IS*Aba1*	+	-	+	IS*Aba1*
GCA_020104975.1	DETAB-E227	+	IS*Aba1*	+	-	+	IS*Aba1*
GCA_020105275.1	DETAB-E246	+	IS*Aba1*	+	-	+	IS*Aba1*
GCA_020105435.1	DETAB-E247	+	IS*Aba1*	+	-	+	IS*Aba1*
GCA_020105835.1	DETAB-E274	+	IS*Aba1*	+	-	+	IS*Aba1*
GCA_020105975.1	DETAB-E29	+	IS*Aba1*	+	-	+	IS*Aba1*
GCA_020106075.1	DETAB-E281	+	IS*Aba1*	+	-	+	IS*Aba1*
GCA_020106275.1	DETAB-E31	+	IS*Aba1*	+	-	+	IS*Aba1*
GCA_020106295.1	DETAB-E302	+	IS*Aba1*	+	-	+	IS*Aba1*
GCA_020106415.1	DETAB-E4	+	IS*Aba1*	+	-	+	IS*Aba1*
GCA_020106515.1	DETAB-E309	+	IS*Aba1*	+	-	+	-
GCA_020106535.1	DETAB-E310	+	IS*Aba1*	+	-	+	-
GCA_020106845.1	DETAB-E337	+	IS*Aba1*	+	-	+	IS*Aba1*
GCA_020107095.1	DETAB-E342	+	IS*Aba1*	+	-	+	IS*Aba1*
GCA_020107135.1	DETAB-E339	+	IS*Aba1*	+	-	+	IS*Aba1*
GCA_020107555.1	DETAB-E364	+	IS*Aba1*	+	-	+	IS*Aba1*
GCA_020108075.1	DETAB-E5	+	IS*Aba1*	+	-	+	IS*Aba1*
GCA_020108135.1	DETAB-E400	+	IS*Aba1*	+	-	+	IS*Aba1*
GCA_020108155.1	DETAB-E171	+	IS*Aba1*	+	-	+	IS*Aba1*
GCA_020108555.1	DETAB-E71	+	IS*Aba1*	+	-	+	IS*Aba1*
GCA_020109495.1	DETAB-E425	+	IS*Aba1*	+	-	+	IS*Aba1*
GCA_020109615.1	DETAB-E157	+	IS*Aba1*	+	-	+	IS*Aba1*
GCA_020109735.1	DETAB-E187	+	IS*Aba1*	+	-	+	IS*Aba1*
GCA_020109835.1	DETAB-E32	+	IS*Aba1*	+	-	+	IS*Aba1*
GCA_020110475.1	DETAB-E123	+	IS*Aba1*	+	-	+	IS*Aba1*
GCA_020110515.1	DETAB-E128	+	IS*Aba1*	+	-	+	IS*Aba1*
GCA_020110955.1	DETAB-E40	+	IS*Aba1*	+	-	+	IS*Aba1*
GCA_020110975.1	DETAB-E41	+	IS*Aba1*	+	-	+	IS*Aba1*
GCA_020111875.1	DETAB-E299	+	IS*Aba1*	+	-	+	IS*Aba1*
GCA_020111935.1	DETAB-E382	+	IS*Aba1*	+	-	+	IS*Aba1*
GCA_020112215.1	DETAB-E81	+	IS*Aba1*	+	-	+	IS*Aba1*
GCA_020112895.1	DETAB-E112	+	IS*Aba1*	+	-	+	IS*Aba1*
GCA_020113755.1	DETAB-E117	+	IS*Aba1*	+	-	+	IS*Aba1*
GCA_020113775.1	DETAB-E327	+	IS*Aba1*	+	-	+	IS*Aba1*
GCA_020114155.1	DETAB-E236	+	IS*Aba1*	+	-	+	IS*Aba1*
GCA_900117405.1	ABE12_M	+	-	+	-	+	IS*Aba1*
GCA_900119395.1	ABE8_07	+	-	+	-	+	IS*Aba26*
GCA_016538365.2	MRSN11835	+	IS*Aba1*	-	-	+	IS*Aba1*
GCF_001640065.1	AB217	+	IS*Aba1*	+	-	+	IS*Aba1*
GCF_001640075.1	AB250	+	IS*Aba1*	+	-	+	IS*Aba1*
GCF_002212105.1	AB552	-	-	+	-	+	IS*Aba1*

SAMEA5593850	Ab174	-	-	-	-	+	-
GCA_009035845.1	ATCC-19606	-	-	-	-	+	-
GCF_008632635	K09-14	-	-	-	-	+	-
GCA_000981405.1	Ab1	-	-	-	-	+	IS*Aba1*

IS, insertion sequences; detected (+); not detected (-).

Table S4. List of antibiotics disk used against Gram-negative and Gram-positive isolates for antibiotic susceptibility screening.

**Gram-negative Bacteria**	**Gram-positive Bacteria**
Ampicillin 10 μg	Benzylpenicillin 10 units
Amoxicillin/Clavulanic acid 20/10 μg	Ampicillin 10 μg
Piperacillin/Tazobactam 100/10 μg	Azithromycin 15 μg
Cefazolin 30 ug	Vancomycin 30 μg
Azithromycin 15 μg	Ciprofloxacin 5 μg
Levofloxacin 5 μg	Clindamycin 2 μg
Cefepime 30 μg	Erythromycin 15 μg
Imipenem 10 μg	Tetracycline 30 μg
Meropenem 10 μg	Imipenem 10 μg
Gentamicin 10 μg	Gentamicin 10 μg
Amikacin 30 μg	Rifampin 5 μg
Trimethoprim/Sulfamethoxazole 1.25/23.75 μg	Trimethoprim/Sulfamethoxazole 1.25/23.75 μg
Ciprofloxacin 5 μg	Cefazolin 30 μg
Chloramphenicol 30 μg	Chloramphenicol 30 μg
Streptomycin 10 μg	
Tetracycline 30 μg	
Piperacillin 100 μg	
Ceftazidime/ Clavulanic acid 30/10 μg	
Kanamycin 30 μg	
